# Efficacy of ketogenic diet on glycemic-lipid metabolism and menstrual regularity in women with polycystic ovary syndrome: a randomized controlled trial

**DOI:** 10.3389/fnut.2026.1920489

**Published:** 2026-09-22

**Authors:** Min Wang, Lingyun Xie, Ling Ma, Cuijun Zhang, Ya Wei, Yulu Wei, Yaru Li, Yang Xu, Jingjing Wang

**Affiliations:** 1 Department of Clinical Nutrition, Yangzhou Maternal and Child Health Care Hospital Affiliated to Yangzhou University, Yangzhou, China; 2 Department of Nutrition, Central Hospital, Tianjin University/Tianjin Third Central Hospital, Tianjin, China

**Keywords:** insulin resistance, ketogenic diet, menstrual regularity, polycystic ovary syndrome, simulated Mediterranean diet

## Abstract

**Introduction:**

Polycystic ovary syndrome (PCOS) is characterized by insulin resistance and menstrual irregularity. Ketogenic diet (KD) improves glycemic-lipid profiles and reduces inflammation, yet its efficacy on menstrual regularity in PCOS remains unclear. This trial evaluated KD effects on glycemic-lipid metabolism and menstrual restoration in PCOS women.

**Methods:**

This single-center, randomized, open-label, parallel-controlled preliminary exploratory trial enrolled 60 Chinese women with PCOS, randomly assigned to ketogenic diet (KD, *n =* 30) or simulated Mediterranean diet (SMD, *n =* 30) for 12 weeks. Total daily caloric intake was consistent between the two groups, based on resting energy expenditure (REE) estimated using the Mifflin-St Jeor equation, with a standardized daily energy deficit of 500 kcal. The macronutrient composition differed structurally: KD provided 70–75% fat, 15–20% protein, and <10% carbohydrates (<50 g/day), whereas SMD provided 30–35% fat, 15–20% protein, and 45–50% carbohydrates. The primary endpoints were change in HOMA-IR and menstrual regularity. Secondary endpoints included anthropometric parameters, glycemic markers, lipid profiles, hormonal levels, and safety parameters. Nutritional ketosis was verified by daily urinary ketone testing and weekly blood ketone monitoring, and dietary adherence was assessed using a standardized compliance score.

**Results:**

This study showed that improvements in body weight, waist circumference, and visceral fat area in PCOS patients were positively correlated with reductions in fasting insulin (FINS) and triglycerides (TG). Compared with SMD, the KD group demonstrated significantly greater improvements in fasting insulin, HOMA-IR, and triglycerides, and these differences remained significant after adjustment for baseline values using analysis of covariance (ANCOVA). Menstrual regularity was restored in 96.7% of KD participants versus 66.7% in the SMD group (*p* = 0.006), with a number needed to treat of 3.3. KD produced substantially greater reductions in body weight (−16.03 vs. −6.03 kg; *p* < 0.0001), BMI (−6.10 vs. −2.23 kg/m^2^; *p* < 0.0001), waist circumference (−17.23 vs. −5.80 cm; *p* < 0.0001), and visceral fat area (−51.27 vs. −13.21 cm^2^; *p* < 0.0001). Neither diet adversely affected hepatic or renal function or protein status; moreover, KD reduced ALT and AST more than SMD. Both diets demonstrated favorable safety profiles with no serious adverse events and 100% completion rate.

**Conclusion:**

During the 12-week short-term intervention, the KD dietary intervention was superior to the SMD dietary intervention in improving insulin resistance and restoring menstrual regularity in women with PCOS. KD is suitable for acute metabolic intervention given its pronounced weight reduction and insulin sensitivity improvement; however, these findings were obtained in a single-center, exploratory trial and require validation in multi-center, larger-scale randomized controlled trials, and its long-term population impact and reproductive outcomes remain to be further investigated. SMD offers anti-inflammatory advantages and safety profiles conducive to long-term health maintenance. Therefore, a sequential strategy of initial KD followed by SMD transition is recommended to achieve sustained metabolic benefits and optimize fertility outcomes.

## 1 Introduction

Polycystic ovary syndrome (PCOS) affects 8–13% of reproductive-aged women worldwide (1). Following a 2026 global consensus, this condition has been formally renamed to polyendocrine metabolic ovarian syndrome (PMOS) (1, 2). This nomenclature change reflects a fundamental reconceptualization of the condition as a multisystem disorder characterized by insulin resistance, hyperandrogenism, and metabolic dysfunction extending far beyond ovarian morphology. PCOS is associated with a substantially elevated risk of type 2 diabetes, dyslipidemia, cardiovascular disease, and infertility, profoundly impacting both metabolic health and quality of life (3, 4). Insulin resistance represents a core pathophysiological feature, present in 50–70% of affected women, driving both metabolic disturbances and reproductive dysfunction. Current management strategies include lifestyle modification, pharmacological interventions such as metformin and oral contraceptives, and symptomatic treatments; however, these approaches often present limited efficacy, adverse effects, and inability to address underlying metabolic abnormalities holistically (5, 6). Consequently, dietary intervention has emerged as a cornerstone of PCOS management, with growing evidence supporting the efficacy of various dietary patterns in improving metabolic and reproductive outcomes (7).

The ketogenic diet (KD), characterized by high fat, moderate protein, and severely restricted carbohydrates, induces nutritional ketosis and fundamentally shifts cellular metabolism from glucose dependency to fatty acid β-oxidation and ketone body production (8). Originally developed for epilepsy treatment in the 1920s, KD has demonstrated therapeutic potential across diverse conditions including type 2 diabetes, obesity, and cardiovascular diseases. Among dietary approaches for PCOS, the Mediterranean diet emphasizes anti-inflammatory polyphenols, time-restricted eating focuses on circadian rhythm restoration (9), and low-glycemic-index approaches modulate postprandial glucose responses (7). Notably, the ketogenic diet stands apart through its unique capacity to induce nutritional ketosis, fundamentally rewiring cellular substrate utilization from glucose dependency to fat oxidation—an effect that directly targets the insulin resistance central to PCOS pathophysiology (8). Accumulating evidence from recent meta-analyses suggests that KD holds considerable therapeutic potential for improving BMI, body composition, glucose metabolism, and hormonal profiles in PCOS (10, 11), although the optimal dietary strategy for metabolic restoration in this population remains to be definitively established.

Individual RCTs have provided preliminary support for KD in PCOS. Sharifi et al. (12) reported that KD significantly reduced BMI, waist circumference, fasting insulin, and testosterone levels, though their study lacked a dietary control group and had a relatively small sample size. Paoli et al. (13) demonstrated that an 8-week KD intervention improved menstrual regularity; however, the absence of a comparator diet and the short intervention duration limit generalizability. These pioneering studies established KD feasibility in PCOS, yet critical methodological limitations remain unaddressed: the lack of head-to-head comparisons with other evidence-based dietary patterns, heterogeneous protocols across trials, small sample sizes, and insufficient intervention durations (10, 14). Notably, no RCT has ever directly compared KD with the Mediterranean diet for managing PCOS, even though the latter is the most widely recommended diet for metabolic health. This trial was designed to address these gaps by conducting the first randomized controlled head-to-head comparison of KD versus simulated Mediterranean diet (SMD) on glycemic-lipid metabolism and menstrual regularity, providing direct evidence to guide personalized dietary recommendations (15).

Despite the accumulating evidence supporting both KD and Mediterranean dietary patterns in PCOS management, no prior study has conducted a head-to-head comparison within a randomized controlled trial framework. KD acts via nutritional ketosis, whereas SMD targets anti-inflammatory and antioxidant pathways (16). These distinct mechanisms likely confer different therapeutic benefits, and a head-to-head trial is warranted. To our knowledge, this exploratory trial represents one of the few randomized controlled studies to directly compare KD with the Mediterranean diet in PCOS management, addressing a critical void in the existing literature. By employing a rigorous parallel-group design with calorie-matched interventions and comprehensive metabolic and reproductive endpoints, this study aims to determine whether KD offers superior acute metabolic efficacy and to provide direct evidence to guide personalized dietary recommendations in the era of the PCOS nomenclature.

## 2 Materials and methods

### 2.1 Study design and ethics

This study was designed as a single-center, randomized, parallel-group, controlled exploratory clinical trial comparing the metabolic and reproductive effects of a KD versus a SMD in overweight and obese women with PCOS. The trial was conducted at the Yangzhou Maternal and Child Health Care Hospital Affiliated to Yangzhou University, Yangzhou, China, between June 2024 and December 2025. The study protocol was reviewed and approved by the institutional Ethics Committee (No. 2024-EC-065) and was conducted in strict accordance with the Declaration of Helsinki. All participants provided written informed consent prior to enrollment after receiving a comprehensive explanation of the study objectives, procedures, potential risks, and benefits. The trial was registered at the Chinese Clinical Trial Registry (ChiCTR2600122054). All study procedures and reporting followed the Consolidated Standards of Reporting Trials (CONSORT) guidelines to guarantee methodological rigor and reproducibility.

### 2.2 Participants

This study enrolled patients with PCOS who presented to the reproductive endocrinology clinic of our hospital between June 2024 and December 2025. Inclusion criteria were age 18–40 years; diagnosis of PCOS per Rotterdam criteria, requiring at least two of the following three features: oligo- or anovulation (menstrual cycles >35 days or <8 per year), clinical or biochemical hyperandrogenism, and polycystic ovarian morphology on transvaginal ultrasound (17); BMI ≥ 24 kg/m^2^; and willingness to strictly adhere to the prescribed dietary protocol throughout the 12-week intervention. Exclusion criteria were pregnancy or lactation; confirmed diabetes or thyroid dysfunction; use of metabolic or reproductive agents within the past 3 months, including oral contraceptives, insulin sensitizers, or anti-androgens; concurrent participation in another clinical trial within the past 6 months; and any medical condition that would compromise safety during dietary intervention. Eligible subjects who provided written informed consent were enrolled in this study.

### 2.3 Randomization and masking

Eligible participants were randomly allocated to either the KD group or the SMD group in a 1:1 ratio using a computer-generated randomization sequence. Allocation concealment was maintained through sequentially numbered, opaque, sealed envelopes that were opened only after enrollment was finalized. Given the nature of the dietary intervention, neither the participants nor the study investigators could be blinded to group assignment. However, laboratory personnel who performed the biochemical and hormonal assays were masked to group allocation to minimize detection bias. All outcome assessors were trained to follow standardized evaluation protocols to ensure consistency and objectivity across both study groups.

### 2.4 Interventions

Participants in the KD group followed a standardized ketogenic dietary protocol providing 70–75% of total daily calories from fat, 15–20% from protein, and less than 10% from carbohydrates, with total carbohydrate intake restricted to fewer than 50 g per day. Daily caloric intake was individualized based on baseline energy requirements and set between 1,200 and 1,500 kcal per day. The dietary plan emphasized healthy fat sources including avocado, nuts, olive oil, and fatty fish, while incorporating moderate protein and strictly limiting carbohydrate-rich foods. Registered dietitians developed individualized weekly meal plans and recipes, and provided structured nutritional counseling to support adherence. Daily urinary ketone testing was performed throughout the intervention period to verify and maintain nutritional ketosis, and urinary ketone–positive days were recorded. In addition, blood ketone levels were monitored weekly in the KD group.

Participants in the SMD group followed a modified Mediterranean dietary pattern adapted for the Chinese population, with caloric intake matched to the KD group at 1,200–1,500 kcal per day. The dietary protocol was designed to simulate the core components of the traditional Mediterranean diet while incorporating locally available foods and cultural preferences. Additional core components included abundant consumption of vegetables and fruits in diverse colors to ensure varied polyphenol intake, whole grains, legumes, nuts, and seeds. Moderate intake of fish and poultry provided omega-3 fatty acids and lean protein, while red meat and processed foods were limited. Wine was excluded to accommodate cultural and dietary preferences of the local population. Registered dietitians delivered nutritional counseling with equivalent frequency and intensity to that provided to the KD group, with particular emphasis on polyphenol-rich food selection and anti-inflammatory cooking methods.

Both groups received weekly nutritional counseling during the initial 4 weeks, transitioning to biweekly counseling for the remainder of the 12-week intervention. All participants were provided with standardized physical activity recommendations of at least 150 min per week of moderate-intensity aerobic activity, consistent with international guidelines for adults. Representative daily menus illustrating typical food choices, portion sizes, and macronutrient composition for each dietary pattern are provided in Supplementary Table S4.

### 2.5 Outcomes

The study employed two co-primary outcomes. The first was the change in homeostatic model assessment for insulin resistance (HOMA-IR) from baseline to week 12. The second was the change in menstrual regularity, defined as the proportion of participants achieving menstrual cycles of 21–35 days in length by the end of the intervention period.

Secondary outcomes included the following measures. Anthropometric parameters comprised body weight, body mass index (BMI), waist circumference, and visceral fat area assessed by bioelectrical impedance analysis. Glycemic parameters included fasting plasma glucose and fasting serum insulin. Lipid profiles included triglycerides, high-density lipoprotein cholesterol, and low-density lipoprotein cholesterol. Hormonal and reproductive measures included total serum testosterone and menstrual recovery rate among participants with baseline amenorrhea. Dietary adherence was assessed using a standardized 5-point Likert scale. Safety parameters included liver function markers, renal function indicators, and complete blood counts.

### 2.6 Assessments

All assessments were conducted at two time points: baseline (week 0, prior to randomization) and at the completion of the 12-week intervention. Anthropometric measurements were performed using standardized protocols. Height was measured to the nearest 0.1 cm with a stadiometer, and body weight was recorded to the nearest 0.1 kg using a calibrated digital scale. Waist circumference was measured at the midpoint between the lowest rib margin and the iliac crest with participants in the standing position after a normal expiration. Blood samples were collected after an overnight fast of at least 10 h for metabolic and hormonal analyses. HOMA-IR was calculated using the formula: (FBG [mmol/L] × FINS [μIU/mL])/22.5 (18). HOMA-IR was selected as a clinically feasible surrogate marker for insulin resistance in this outpatient-based trial; although the euglycemic-hyperinsulinemic clamp represents the gold standard for measuring insulin sensitivity, its invasive nature, resource requirements, and participant burden make it impractical for dietary intervention trials, and HOMA-IR has demonstrated good correlation with clamp-derived measures in validation studies (19, 20). Menstrual history and cycle characteristics were documented through standardized questionnaires administered by trained research personnel at each assessment visit.

### 2.7 Statistical analysis

SPSS (version 26.0) and GraphPad Prism (version 8.2.1) were used for data analyses and visualization. Variable data distribution was evaluated using the Shapiro–Wilk test. Normally and non-normally distributed data are expressed as means ± standard deviations and medians with interquartile ranges (IQRs), respectively; categorical variables are expressed as counts and percentages and were compared using the *χ*^2^ test or Fisher’s exact test, as appropriate. Correlation analysis was conducted using a Pearson’s correlation coefficient and simple linear regression. Within-group changes were assessed using paired *t*-tests, and between-group comparisons were performed using independent t-tests. One-way analysis of variance (ANOVA) was used for multiple samples. For variables with significant skewness (fasting insulin and HOMA-IR; Shapiro–Wilk *p* < 0.05), natural logarithmic (ln) transformation was applied prior to parametric testing, and non-parametric tests (Wilcoxon signed-rank test for within-group comparisons and Mann–Whitney *U* test for between-group comparisons) were additionally performed on untransformed data as sensitivity analyses. Given the baseline imbalances in menstrual regularity and triglycerides between groups, analysis of covariance (ANCOVA) with baseline values as covariates was performed for the primary outcomes and triglycerides (Supplementary Table S1); both unadjusted and baseline-adjusted results are reported. Repeated-measures mixed-effects models (time × group interaction) were fitted as a further sensitivity analysis (Supplementary Table S2). The absolute risk reduction (ARR) and number needed to treat (NNT) with 95% confidence intervals were calculated for menstrual regularity restoration. A post-hoc power analysis was performed for the primary outcome of menstrual regularity (Supplementary Table S3). *p* < 0.05 was considered to indicate statistical significance.

## 3 Results

### 3.1 Participant characteristics

A total of 75 women were assessed for eligibility, of whom 60 participants meeting the inclusion criteria were randomized into either KD group (*n* = 30) or the SMD group (*n* = 30). The CONSORT diagram illustrating the flow of participants through each stage of the trial is presented in Figure 1. All 60 participants completed the full 12-week dietary intervention, yielding a 100% completion rate with no dropouts and no protocol deviations.

**Figure 1:**
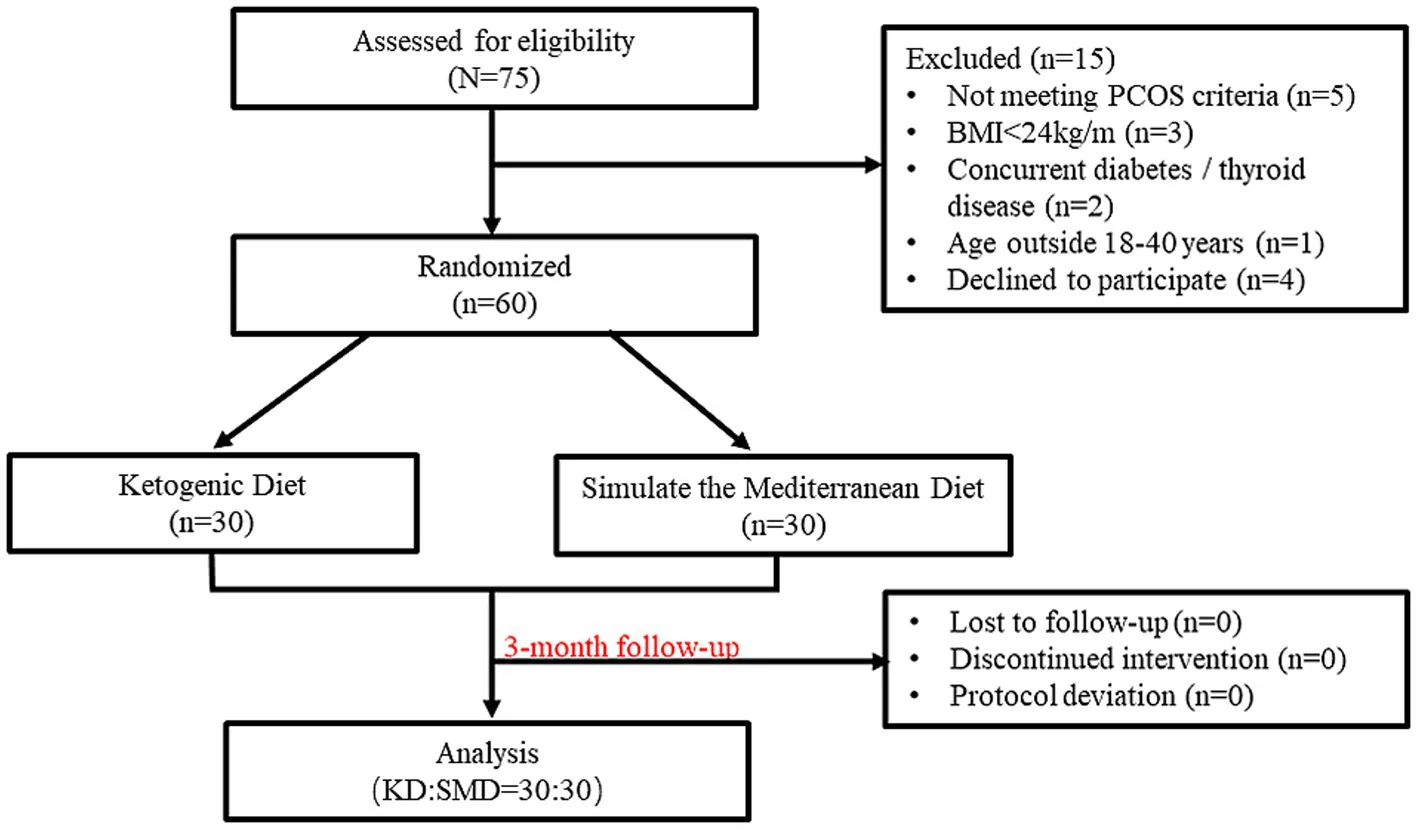
CONSORT flow diagram. Flow of participants through the randomized controlled trial comparing KD versus SMD in women with PCOS. All participants completed the 12-week intervention with no dropouts.

Baseline demographic and clinical characteristics are summarized in Table 1. The two groups were well-balanced in age, anthropometric parameters, glycemic markers, HOMA-IR, testosterone, and lipid profiles except triglycerides. Notably, despite randomization, regular menstruation at baseline was significantly less prevalent in the KD group (10.0%, 3/30) compared with the SMD group (40.0%, 12/30; Fisher’s exact test *p* = 0.015), while amenorrhea rates were comparable (43.3% *vs*. 50.0%; *p* = 0.796). Baseline triglyceride levels were also higher in the KD group (1.93 ± 0.79 vs. 1.48 ± 0.65 mmol/L; *p* = 0.021). These baseline imbalances were addressed using ANCOVA with baseline values as covariates and prespecified sensitivity analyses (see Sections 2.7 and 3.3).

**Table 1:** Baseline characteristics of study participants.

Characteristic	KD Group (*n =* 30)	SMD Group (*n =* 30)	*p*-value
Age (years)	28.97 ± 4.31	28.17 ± 6.37	0.571
Height (cm)	161.82 ± 6.08	162.07 ± 5.73	0.870
Weight (kg)	90.67 ± 14.53	86.58 ± 17.91	0.336
BMI (kg/m^2^)	34.60 ± 5.14	32.84 ± 5.65	0.213
Waist circumference (cm)	106.13 ± 11.34	99.63 ± 14.69	0.060
Visceral fat area (cm^2^)	135.07 ± 39.84	114.64 ± 44.73	0.071
Fasting glucose (mmol/L)	5.75 ± 0.95	5.88 ± 1.51	0.694
Fasting insulin (μU/mL)	32.24 ± 14.26	29.47 ± 24.76	0.598
HOMA-IR	8.68 ± 5.00	8.86 ± 12.14	0.941
Testosterone (nmol/L)	0.51 ± 0.25	0.45 ± 0.44	0.514
Triglycerides (mmol/L)	1.93 ± 0.79	1.48 ± 0.65	0.021^*^
HDL-C (mmol/L)	1.11 ± 0.22	1.21 ± 0.31	0.128
LDL-C (mmol/L)	3.26 ± 0.62	3.04 ± 0.98	0.312
Regular menstruation, n (%)	3 (10.0)	12 (40.0)	0.015^*^
Amenorrhea, n (%)	13 (43.3)	15 (50.0)	0.796

*p < 0.05.

Table 2 shows the macronutrient composition of the two dietary interventions. Total energy intake was comparable between KD and SMD. Protein content was well-matched in both absolute and relative terms. However, the two diets differed markedly in carbohydrate and fat composition: KD provided substantially lower carbohydrate and higher fat compared to SMD.

**Table 2:** Macronutrient composition of dietary interventions.

Parameter	KD (*n =* 30)	SMD (*n =* 30)	*P*-value
Total Energy intake (kcal/day)	1397.42 ± 106.19	1361.83 ± 137.32	0.266
Protein (kcal)	251.54 ± 19.63	245.13 ± 18.27	0.196
Fat (kcal)	1006.14 ± 81.05	435.78 ± 35.57	<0.0001^****^
Carbohydrate (kcal)	139.74 ± 9.99	680.91 ± 56.38	<0.0001^****^
Protein (g)	62.88 ± 4.91	61.28 ± 4.57	0.197
Fat (g)	111.79 ± 9.01	48.42 ± 3.95	<0.0001^****^
Carbohydrate (g)	34.94 ± 2.50	170.23 ± 14.10	<0.0001^****^
Protein (%)	18.00 ± 2.00	18.00 ± 2.00	1.000
Fat (%)	72.00 ± 2.00	32.00 ± 2.00	<0.0001^****^
Carbohydrate (%)	10.00 ± 2.00	50.00 ± 2.00	<0.0001^****^

****p < 0.0001.

### 3.2 Anthropometric outcomes

The associations between changes in anthropometric parameters and changes in metabolic outcomes are visualized in Figure 2A. Improvements in weight, waist circumference, and visceral fat area were positively correlated with reductions in fasting insulin (FINS) and triglycerides (TG).

**Figure 2:**
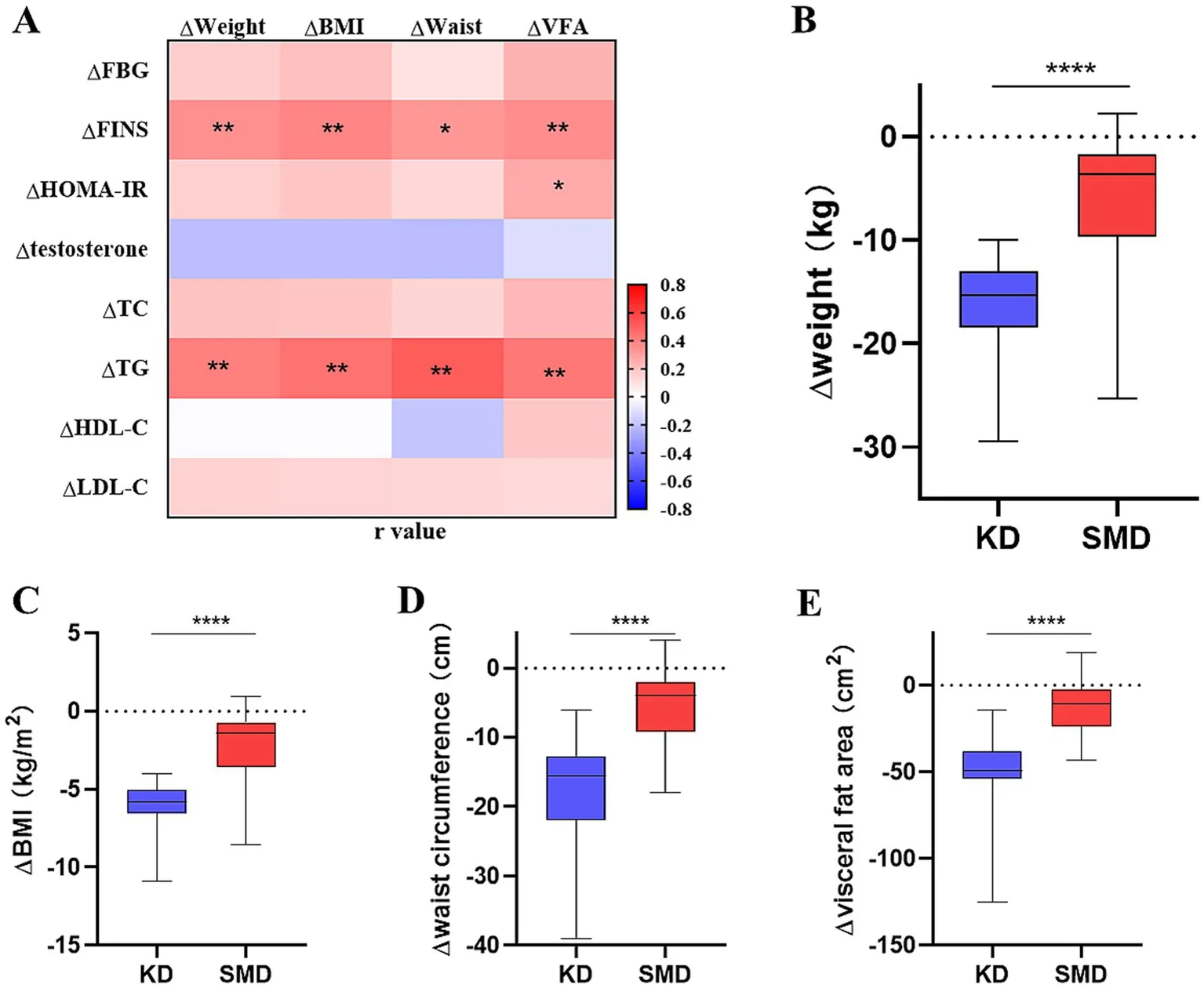
Anthropometric changes and correlation analysis. **(A)** Correlation analysis of changes in anthropometric and metabolic parameters. Pearson *r* = (−1, 1). Changes in body weight **(B)**, BMI **(C)**, waist circumference **(D)** and visceral fat area **(E)** between-group comparisons. KD, ketogenic diet; SMD, simulated Mediterranean diet; FBG, fasting blood glucose; FINS, fasting insulin; HOMA-IR, Homeostatic Model Assessment for Insulin Resistance; TC, total cholesterol; TG, triglycerides; HDL-C, high-density lipoprotein cholesterol; LDL-C, low-density lipoprotein cholesterol; VFA, visceral fat area. *p < 0.05, **p < 0.01, ***p < 0.001, ****p < 0.0001.

The KD group achieved significantly greater reductions across all anthropometric measures compared with the SMD group. Body weight decreased by −16.03 kg in the KD group versus −6.03 kg in the SMD group (Figure 2B). BMI decreased by −6.10 kg/m^2^ in the KD group compared with −2.23 kg/m^2^ in the SMD group (Figure 2C). Waist circumference was reduced by −17.23 cm in the KD group versus −5.80 cm in the SMD group (Figure 2D). Visceral fat area decreased by −51.27cm^2^ in the KD group compared with −13.21 cm^2^ in the SMD group (Figure 2E). The KD group consistently demonstrated approximately two- to four-fold greater improvements in anthropometric measures relative to the SMD group. The largest absolute reduction was observed in visceral fat area, which decreased by more than 50 cm^2^ in the KD group.

### 3.3 Primary outcomes

The primary outcomes of insulin resistance, as measured by HOMA-IR, and menstrual regularity are presented in Figure 3. Following the 12-week intervention, HOMA-IR decreased significantly in both the KD and SMD groups. In the KD group, HOMA-IR declined substantially from 8.68 at baseline to 2.45 at week 12, representing a mean change of −6.25. In the SMD group, HOMA-IR decreased from 8.86 at baseline to 5.75 at week 12, with a mean change of −3.11. The between-group comparison revealed that the magnitude of HOMA-IR reduction was significantly greater in the KD group than in the SMD group. The KD group achieved a 72.0% relative reduction in HOMA-IR compared with a 35.1% relative reduction in the SMD group. Because fasting insulin and HOMA-IR were non-normally distributed (Shapiro–Wilk *p* < 0.05), between-group comparisons were performed on ln-transformed values and were confirmed by Mann–Whitney *U* tests on untransformed data, which yielded consistent results (all *p* < 0.05). After adjustment for baseline values using ANCOVA, the between-group difference in HOMA-IR reduction remained statistically significant (adjusted *p* < 0.001; Supplementary Table S1); likewise, the between-group difference in triglyceride reduction remained significant after baseline adjustment (adjusted *p* = 0.003; Supplementary Table S1). Similarly, the group effect on HOMA-IR remained significant after additional adjustment for the amount of weight loss (*F* = 9.94, *p* = 0.003), with weight loss itself showing no significant association (*p* = 0.781). One KD participant had a missing baseline HOMA-IR measurement; analyses involving HOMA-IR were therefore performed on 59 participants.

**Figure 3:**
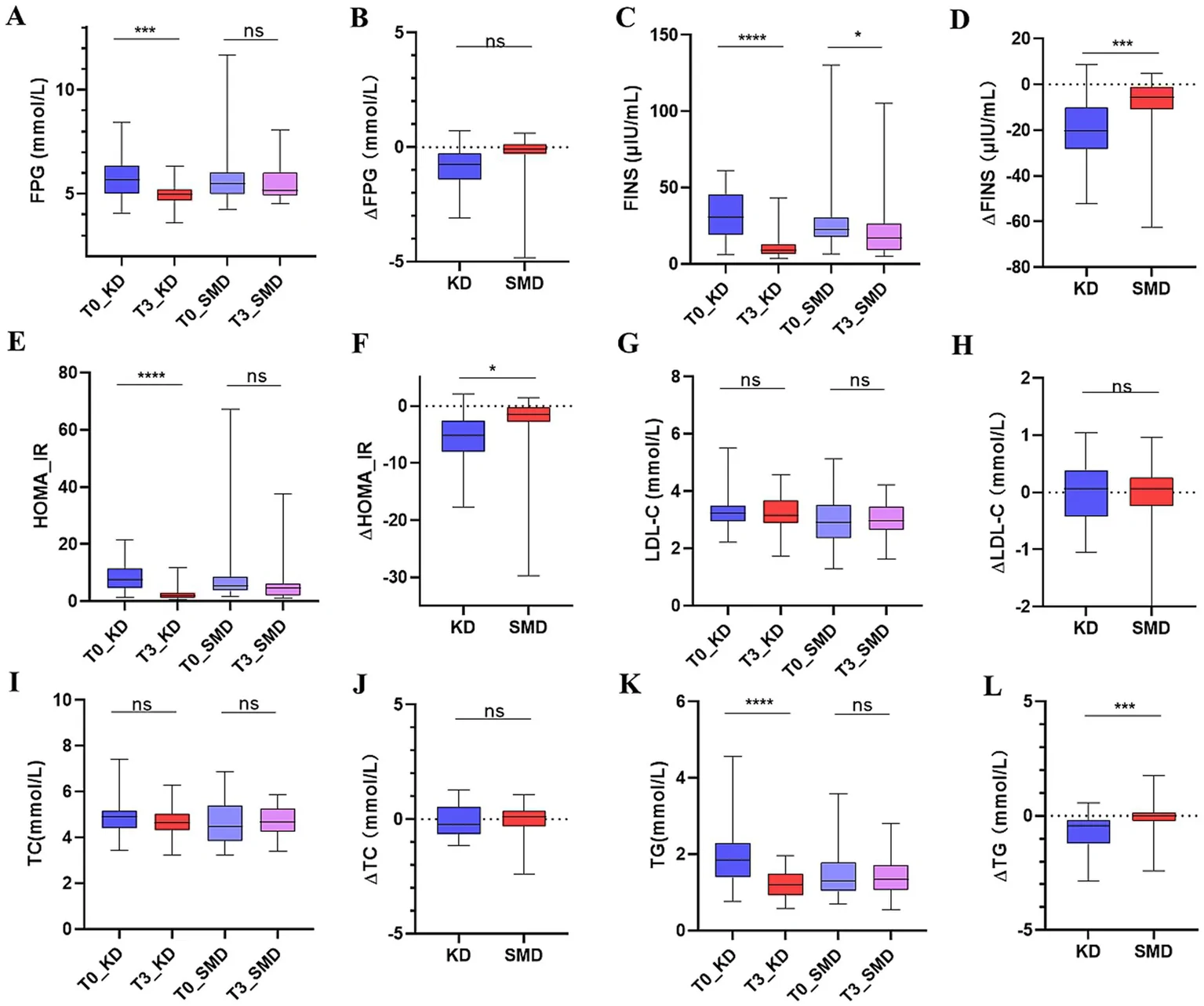
Metabolic parameter changes. **(A,C,E,G,I,K)** FPG, FINS, HOMA-IR, LDL-C, TC, and TG at baseline (T0) and week 12 (T3). **(B,D,F,H,J,L)** Changes in FPG, FINS, HOMA-IR, LDL-C, TC, and TG between-group comparisons. *p < 0.05, ***p < 0.001, ****p < 0.0001; ns = not significant.

The proportion of participants with regular menstruation increased from 10.0% at baseline to 96.7% at week 12 in the KD group. In comparison, the SMD group showed a more modest increase from 40.0% at baseline to 66.7% at week 12 (Figure 4A). After adjustment for baseline menstrual regularity using ANCOVA, the KD group demonstrated significantly greater improvement in menstrual regularity than the SMD group (*F* = 22.82, *p* < 0.001; Supplementary Table S1). A further sensitivity analysis adjusting for the amount of weight loss in addition to baseline values confirmed that the group effect on menstrual regularity remained significant (*F* = 15.76, *p* < 0.001), whereas weight loss itself was not a significant predictor (*p* = 0.615). A sensitivity analysis excluding the 15 participants with regular menstruation at baseline (3 in KD, 12 in SMD) confirmed that menstrual regularity was restored in a significantly higher proportion of participants in the KD group (96.3%, 26/27) than in the SMD group (44.4%, 8/18; *χ*^2^ = 15.72, *p* < 0.001), indicating that the observed effect was not driven by the baseline imbalance. The absolute risk reduction for menstrual regularity restoration was 30.0% (95% CI: 10.6–48.1%), corresponding to a number needed to treat (NNT) of 3.3 (95% CI: 2.1–9.4). And 92.3% (12 of 13) of participants with amenorrhea in the KD group recovered menstruation during the intervention period, compared with only 33.3% (5 of 15) of participants with amenorrhea in the SMD group (Figure 4B). Total testosterone levels and their changes from baseline did not differ significantly between groups or over time (Figures 4C,D).

**Figure 4:**
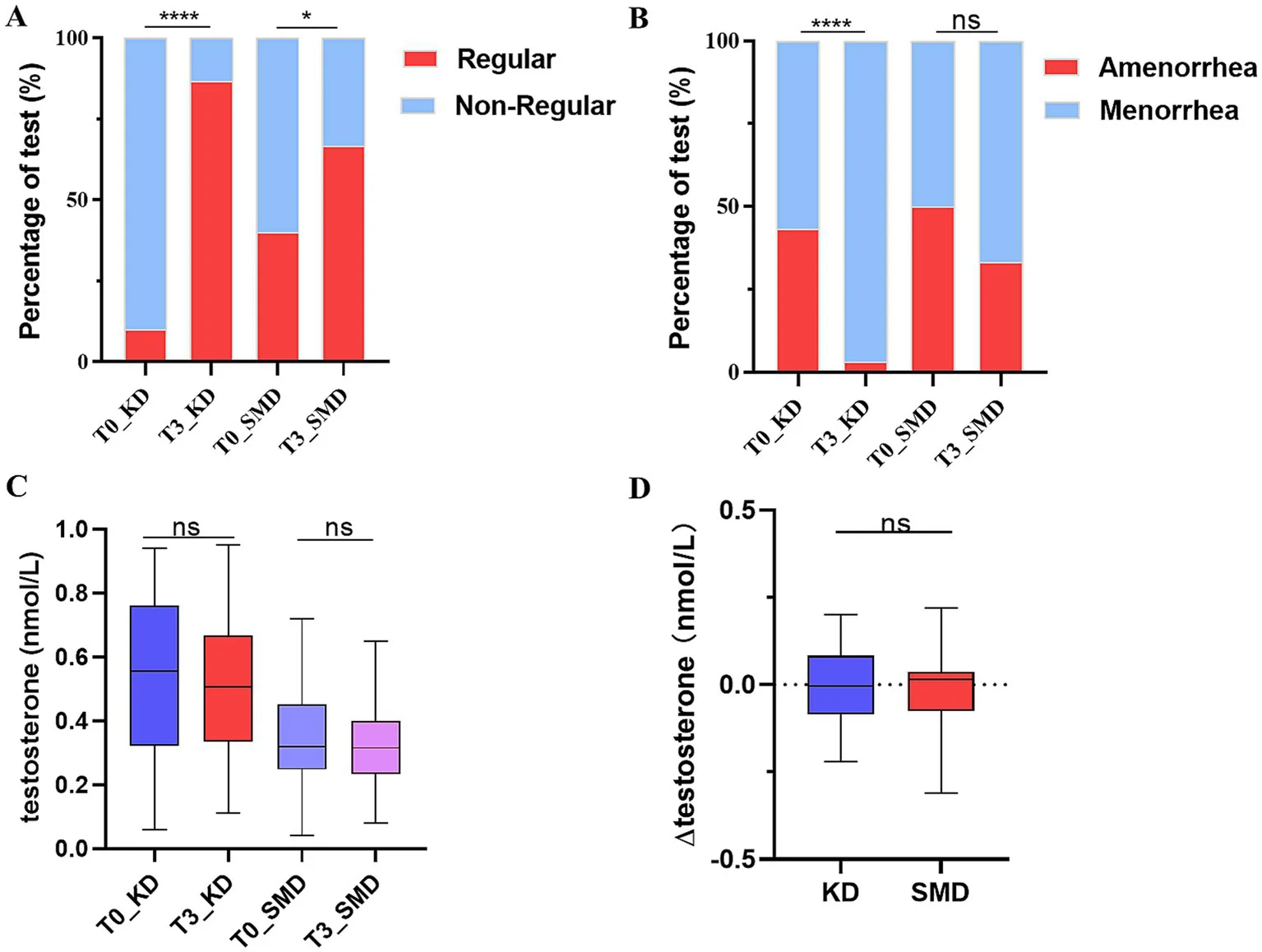
Menstrual outcomes and hormonal changes. **(A)** Percentage of participants with regular and non-regular menstruation at T0 and T3 for KD and SMD groups. **(B)** Percentage of participants with amenorrhea and menstrual recovery among those with baseline amenorrhea. **(C)** Serum testosterone levels at T0 and T3. **(D)** Changes in testosterone between groups. *p < 0.05, ****p < 0.0001; ns = not significant.

### 3.4 Safety and adherence

The safety profiles of the KD and SMD interventions across hepatic, renal, and nutritional parameters are shown in Figure 5. Both groups showed no significant adverse effects on liver function, kidney function, or nutritional status after the 12-week intervention. In addition, levels of AST and ALT decreased significantly from T0 to T3 in the KD group, with no significant change in the SMD group.

**Figure 5:**
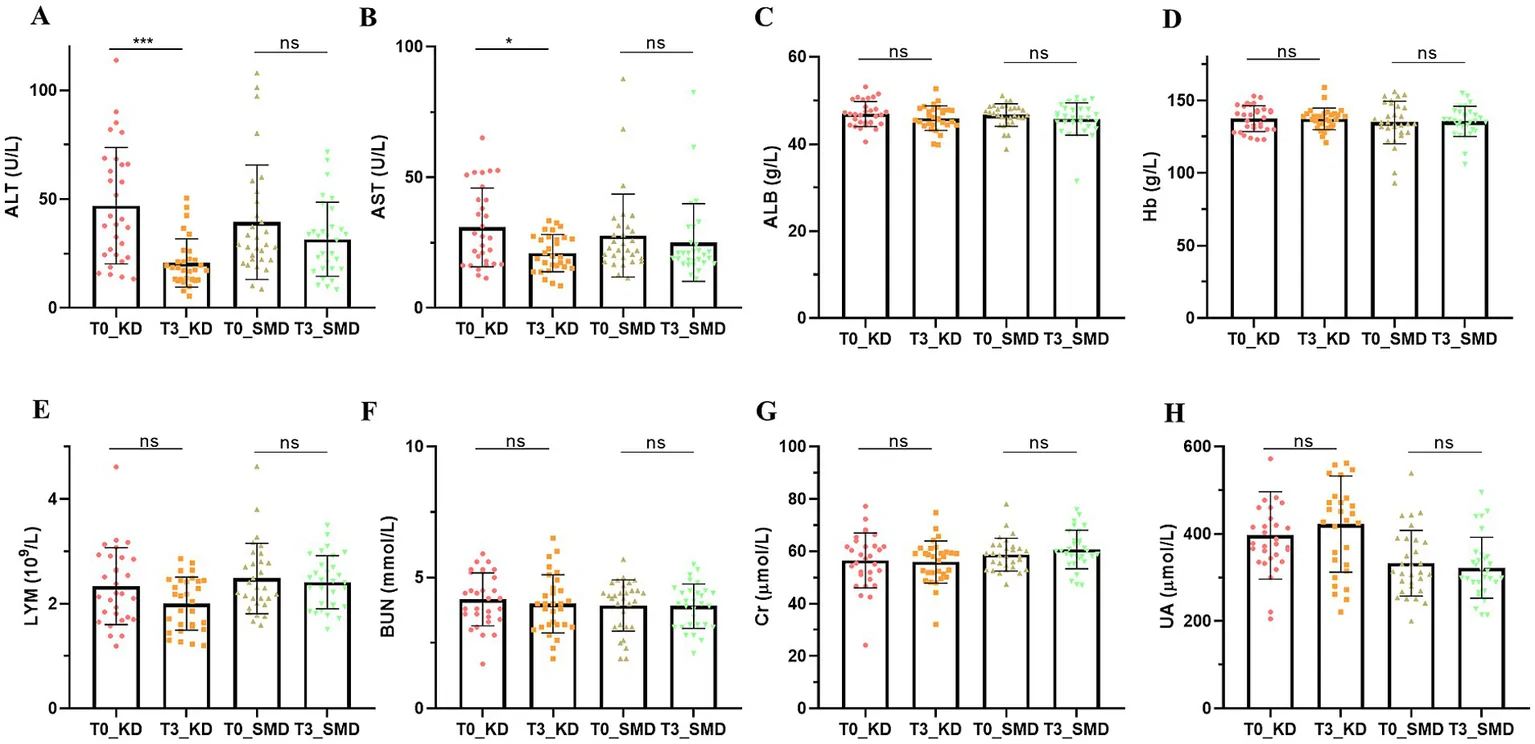
Safety parameters. **(A–H)** ALT, AST, ALB, Hb, LYM, BUN, Cr, UA at T0 and T3. ALT, alanine aminotransferase; AST, aspartate aminotransferase; ALB, albumin; Hb, hemoglobin; LYM, lymphocytes; BUN, blood urea nitrogen; Cr, creatinine; UA, uric acid. *p < 0.05, ***p < 0.001; ns = not significant.

No serious adverse events were reported by any participant during the intervention period. Mild transient adverse events occurred in a subset of participants in the KD group, including constipation (*n* = 3) and headache (*n* = 2); all such events resolved spontaneously within 2 weeks without requiring discontinuation of the dietary intervention or additional medical treatment. No participant in the SMD group reported any adverse events during the study period. These findings demonstrate that both dietary interventions were well tolerated over the 12-week duration.

Dietary adherence, assessed throughout the intervention period using standardized compliance scoring on a scale from 0 to 5, was significantly higher in the KD group than in the SMD group (4.70 ± 0.47 vs. 2.80 ± 1.00; *p* < 0.001). In the KD group, weekly blood ketone monitoring showed a mean ketone concentration of 2.38 ± 0.46 mmol/L (range 1.50–3.20 mmol/L), with all participants maintaining nutritional ketosis (≥0.5 mmol/L) throughout the intervention. Daily urinary ketone testing was positive on 80.5 ± 10.2 of approximately 90 monitored days (mean positivity rate 91.5% ± 2.7%), corroborating effective implementation of the ketogenic protocol.

## 4 Discussion

### 4.1 Principal findings

This exploratory RCT represents the first head-to-head comparison of KD versus SMD in women with PCOS. Our findings demonstrate that KD produced statistically and clinically greater improvements across the primary and key secondary metabolic endpoints. Specifically, KD achieved a significantly greater reduction in HOMA-IR, a markedly higher rate of menstrual regularity restoration, and substantially greater weight loss. Importantly, these between-group differences remained significant after adjustment for baseline imbalances using ANCOVA and in sensitivity analyses excluding participants with regular menstruation at baseline, supporting the robustness of the findings. The superior short-term efficacy of KD is biologically plausible given that severe carbohydrate restriction rapidly depletes hepatic glycogen stores, induces nutritional ketosis, suppresses insulin secretion, and redirects substrate utilization toward fat oxidation, thereby directly targeting the insulin resistance central to PCOS pathophysiology (3, 4). However, the precise molecular mechanisms underlying the restoration of menstrual regularity in the KD group remain to be elucidated. Previous literature has suggested potential roles for β-hydroxybutyrate (BHB)-mediated signaling—including NLRP3 inflammasome inhibition, attenuation of oxidative stress, and gut microbiota remodeling (21–23)—as well as for CYP17A1 inhibition and increased sex hormone-binding globulin (SHBG) in ketogenic diet-mediated hormonal changes. These mechanistic pathways were not directly measured in the present study and should be regarded as hypotheses for future investigation rather than conclusions supported by our data.

The magnitude of KD-induced improvements in our trial exceeds pooled estimates from recent meta-analyses (10, 11). This enhanced efficacy likely reflects our strict dietary monitoring with objective ketone verification, the well-formulated KD with precise macronutrient ratios, and the 12-week intervention duration during which acute ketotic effects are maximal. The remarkable 96.7% menstrual restoration rate—substantially exceeding prior reports of 50–80% (13, 24)—is encouraging; whether it is mediated by ketone-dependent hormonal effects, the substantial weight loss achieved, or their combination cannot be determined from the present data and warrants mechanistic investigation.

The 92.3% restoration rate among participants with baseline amenorrhea is particularly noteworthy, as this population is typically refractory to non-pharmacological interventions. Exogenous ketone supplementation has been reported to acutely lower androgen and glucose levels in women with PCOS (25), and a brief clinical report suggested that KD may improve fertility potential in this population (26). These external findings offer candidate explanations for our observations, but because serum ketones were not correlated with hormonal outcomes in the present study and SHBG, LH, and ovarian steroidogenic enzyme activity were not measured, any mechanistic attribution remains speculative.

### 4.2 Mechanistic considerations: hypotheses for future research

While KD demonstrated superior acute metabolic efficacy, the SMD may confer distinct advantages rooted in its rich content of bioactive polyphenolic compounds from extra-virgin olive oil, vegetables, and legumes (16, 27). Experimental and clinical studies indicate that hydroxytyrosol, oleuropein, and oleocanthal exert anti-inflammatory actions through NF-κB signaling inhibition with subsequent reduction of IL-6 and TNF-α, and olive oil interventions have been shown to improve inflammatory and endothelial markers (28). Polyphenolic compounds may also activate sirtuin pathways and the Nrf2 antioxidant response system, with potential anti-senescence effects (29). In addition, Mediterranean dietary patterns have been associated with favorable gut microbial profiles (22), and Mediterranean diet quality scores have been inversely associated with PCOS risk (30). It must be emphasized, however, that inflammatory biomarkers, polyphenol intake, and gut microbiota composition were not measured in the present trial; the anti-inflammatory advantages attributed to the SMD are therefore inferred from established literature rather than directly validated by our data.

The divergent efficacy profiles of the two diets suggest complementary clinical roles. KD functions as an acute metabolic intervention through nutritional ketosis and substrate switching, producing rapid improvements in body weight and insulin sensitivity, whereas the Mediterranean diet confers anti-inflammatory effects and better long-term adherence in prior comparative studies (15, 31, 32). The pilot study by Mavropoulos et al. (33) first established KD feasibility in women with PCOS, and long-term follow-up of the PREDIMED trial confirmed sustained cardiovascular benefits of the Mediterranean diet (34). These dietary approaches target different but interconnected pathophysiological domains and may be optimally deployed in sequence rather than as alternatives: initial KD for rapid metabolic correction and menstrual restoration, followed by SMD transition for sustained anti-inflammatory protection and cardiovascular risk reduction (35).

The 12-week study duration likely favored the acute metabolic effects of KD, during which nutritional ketosis produces maximal substrate switching and insulin suppression. A longer intervention period might reveal cumulative advantages of SMD, as anti-inflammatory and gut microbiome remodeling effects require sustained exposure to polyphenolic compounds and dietary fiber. The observation that both dietary approaches produced comparable changes in serum testosterone—despite differential effects on body weight and insulin resistance—indicates that distinct pathways may converge on androgen homeostasis in PCOS. It has been hypothesized that KD reduces androgens primarily through insulin-mediated mechanisms (reduced insulin-driven ovarian androgen production and increased hepatic SHBG synthesis) and possibly through direct BHB-mediated suppression of ovarian steroidogenesis, whereas the SMD may act through anti-inflammatory modulation of ovarian theca cell function (4, 25). Because testosterone did not change significantly in either group in the present trial, and SHBG, LH, and CYP17A1 activity were not measured, these convergent-mechanism interpretations should be considered hypotheses to be tested in future studies with comprehensive hormonal and inflammatory profiling.

### 4.3 Potential confounding factors

We acknowledge that the observed improvements in the KD group cannot be solely attributed to ketosis *per se*. The KD group achieved approximately 10 kg greater weight loss and demonstrated significantly better dietary adherence (4.70 *vs*. 2.80) than the SMD group; the superior adherence likely reflects rapid symptomatic improvements reinforcing motivational commitment. These differences may reflect a greater caloric deficit, enhanced dietary compliance, or the combined metabolic effects of these factors, rather than ketosis alone. Both groups were designed with isocaloric targets (1,200–1,500 kcal/day) to minimize caloric differences; however, actual intake may have varied with differential adherence. Accordingly, the present findings should be interpreted as a comparison of two dietary interventions rather than of ketosis in isolation. Future studies should employ matched weight-loss designs or include ketone-supplementation arms to isolate the specific effects of ketosis.

### 4.4 Safety, strengths and limitations

Both interventions demonstrated reassuring safety profiles with 100% completion rates and no serious adverse events. Notably, KD significantly improved hepatic enzyme markers (ALT and AST reductions), consistent with evidence that well-formulated KD ameliorates hepatic steatosis rather than exerting hepatotoxic effects (32). Renal function remained stable in both groups. For longer-term safety context, studies of ketogenic interventions followed by gradual carbohydrate reintroduction have reported sustained benefits without safety concerns (24). These collective safety data support KD as a short-to-medium term metabolic intervention in women with PCOS when implemented with appropriate medical supervision.

Despite the limitations discussed below, this trial has several strengths that support the internal validity of the findings: the randomized controlled design with allocation concealment; the first head-to-head comparison of two distinct, calorie-matched dietary patterns in PCOS; objective verification of nutritional ketosis by weekly blood ketone monitoring and daily urinary ketone testing (positivity rate 91.5%); the use of both subjective (menstrual diary) and objective (biochemical) outcome measures; and the use of baseline-adjusted analyses (ANCOVA) with multiple sensitivity analyses. A post-hoc power analysis indicated that the achieved sample size provided 92% power to detect the observed effect size for menstrual regularity (Cohen’s *h =* 0.86), supporting the adequacy of the sample for the primary outcome (Supplementary Table S3).

This trial has several limitations. First, the single-center design, conducted at the Yangzhou Maternal and Child Health Care Hospital, may limit the generalizability of the findings to other healthcare settings with different demographic and socioeconomic characteristics. Second, the modest sample size (*n =* 60), although adequate for the primary analysis and supported by the post-hoc power analysis, may limit statistical power for subgroup analyses and the detection of smaller effect sizes. Third, due to the distinct nature of the dietary interventions, blinding of participants and investigators was not feasible; this open-label design introduces potential performance and detection bias, which we sought to mitigate by using objective biochemical outcomes and standardized menstrual diary recording with laboratory personnel masked to group allocation. Fourth, the cohort was restricted to Chinese women aged 18–40 years residing in the Yangzhou region, and the findings may not be directly generalizable to other ethnicities, age groups, or geographic regions with different dietary habits and genetic backgrounds. Fifth, the 12-week duration precludes assessment of long-term outcomes, including sustained menstrual regularity, fertility parameters, and pregnancy rates. Sixth, inflammatory biomarkers (IL-6, TNF-α, CRP) and detailed polyphenol intake were not measured, limiting direct biochemical validation of the anti-inflammatory mechanisms attributed to the SMD; moreover, a formal Mediterranean diet adherence score (e.g., the Mediterranean Diet Score) was not employed, and adherence in the SMD group was assessed by compliance scoring and dietary-record review only; these measurements should be incorporated in future trials. Seventh, the reliance on HOMA-IR rather than clamp-derived insulin sensitivity measurements may have limited our ability to detect subtle differences in insulin action between the two dietary interventions, although HOMA-IR correlates well with clamp-derived measures (19, 20). Eighth, despite randomization, baseline imbalances in menstrual regularity and triglycerides occurred; although we addressed these through ANCOVA adjustment and sensitivity analyses, we cannot completely exclude residual confounding. Future research should focus on sequential dietary regimen benefits, optimal transition timing between KD and SMD, mechanistic studies incorporating hormonal (SHBG, LH) and inflammatory profiling, and longer-term reproductive outcomes.

## 5 Conclusion

This randomized controlled exploratory trial provides direct evidence for dietary management in women with PCOS. A 12-week ketogenic dietary intervention achieved superior improvements in insulin resistance and menstrual regularity compared with a simulated Mediterranean dietary intervention, serving as an effective strategy for acute-phase metabolic intervention; these findings reflect the comparison of two dietary interventions and do not isolate the specific contribution of ketosis per se. To our knowledge, this study represents one of the few head-to-head RCTs comparing KD with the Mediterranean diet in PCOS, addressing a critical gap in the existing literature where prior investigations were limited by the absence of dietary comparators, heterogeneous protocols, and small sample sizes. However, the single-center exploratory design, the long-term population effects, reproductive outcomes, and safety profile of KD require further investigation. Future research should include multi-center, larger-scale RCTs involving diverse populations; incorporate inflammatory biomarker (IL-6, TNF-α, CRP) measurements and clamp-derived assessment of insulin sensitivity; test the mechanistic hypotheses proposed here (including the roles of BHB, SHBG, and CYP17A1); and evaluate the long-term benefits of sequential dietary regimens—initial KD for acute metabolic correction followed by SMD transition for sustained maintenance—and the optimal timing for transition between interventions.

## Data Availability

The original contributions presented in the study are included in the article/Supplementary material, further inquiries can be directed to the corresponding authors.
