# Supplementary material for: Efficacy of ketogenic diet on glycemic-lipid metabolism and menstrual regularity in women with polycystic ovary syndrome: a randomized controlled trial
Source: Front Nutr. 2026 Sep 22;13:1920489. doi: 10.3389/fnut.2026.1920489 (PMC13638353; doi:10.3389/fnut.2026.1920489)
Supplement: Supplementary file 1 [file Table_1.docx]

# Supplementary Material

**Manuscript:** Efficacy of Ketogenic Diet on Glycemic-Lipid Metabolism and Menstrual Regularity in Women with Polycystic Ovary Syndrome: A Randomized Controlled Trial

**Journal:** Frontiers in Nutrition

## Supplementary Table S1. Baseline-Adjusted Between-Group Comparisons (ANCOVA) for Primary and Key Secondary Outcomes

| Outcome | n | KD adjusted mean | SMD adjusted mean | Adjusted difference (KD − SMD) | F | p value |
| --- | --- | --- | --- | --- | --- | --- |
| Menstrual regularity (proportion) | 60 | 1.029 | 0.605 | 0.424 | 22.82 | < 0.0001^****^ |
| HOMA-IR | 59 | 2.472 | 5.709 | −3.236 | 21.39 | < 0.0001^****^ |
| Fasting insulin (ln-transformed) | 60 | 2.125 | 2.873 | −0.747 | 30.31 | < 0.0001^****^ |
| Fasting plasma glucose | 59 | 4.968 | 5.496 | −0.528 | 11.26 | 0.001^**^ |
| Triglycerides | 60 | 1.154 | 1.503 | −0.350 | 9.55 | 0.003^**^ |
| Body weight (kg) | 60 | 73.012 | 82.188 | −9.176 | 62.24 | < 0.0001^****^ |
| Menstrual regularity (additionally adjusted for weight loss) | 60 | 1.048 | 0.586 | 0.462 | 15.76 | < 0.0001^****^ |
| HOMA-IR (additionally adjusted for weight loss) | 59 | 2.567 | 5.618 | −3.051 | 9.94 | 0.003^**^ |

*Notes:* Analysis of covariance (ANCOVA) was performed with the week-12 value as the dependent variable, group (KD vs. SMD) as the fixed factor, and the corresponding baseline value as the covariate. Adjusted means are least-squares means estimated at the mean baseline value. Fasting insulin was analyzed on natural logarithm–transformed values because of skewed distribution. Menstrual regularity was analyzed as a binary outcome (1 = regular, 0 = non-regular) using a linear probability model. The last two rows show sensitivity models additionally adjusted for the amount of weight loss (change in body weight from baseline to week 12) as a second covariate. One KD participant had a missing baseline HOMA-IR and fasting glucose measurement (n = 59 for these outcomes). KD, ketogenic diet; SMD, simulated Mediterranean diet. *p < 0.05, **p < 0.01, ***p < 0.001, ****p < 0.0001.

## Supplementary Table S2. Repeated-Measures Mixed-Effects Models (Time × Group Interaction) for Primary and Secondary Outcomes

| Outcome | n | Time effect β (p value) | Group effect β (p value) | Time × Group interaction β (SE) | p value |
| --- | --- | --- | --- | --- | --- |
| HOMA-IR | 59 | −3.107 (p = 0.002**) | −0.181 (p = 0.925) | −3.139 (1.457) | 0.031^*^ |
| Fasting insulin (ln-transformed) | 60 | −0.349 (p < 0.001***) | 0.205 (p = 0.191) | −0.819 (0.140) | < 0.0001^****^ |
| Fasting plasma glucose | 59 | −0.357 (p = 0.049*) | −0.131 (p = 0.624) | −0.444 (0.259) | 0.087 |
| Triglycerides | 60 | −0.040 (p = 0.741) | 0.444 (p = 0.004**) | −0.671 (0.170) | < 0.0001^****^ |
| Body weight | 60 | −6.027 (p < 0.001***) | 4.090 (p = 0.285) | −10.003 (1.402) | < 0.0001^****^ |
| Menstrual regularity | 60 | 0.267 (p < 0.001***) | −0.300 (p = 0.002**) | 0.600 (0.102) | < 0.0001^****^ |

*Notes:* Linear mixed-effects models with a random intercept for each participant were fitted by maximum likelihood. Group was coded as KD = 1, SMD = 0; time was coded as week 12 = 1, baseline = 0. The time × group interaction term tests whether the change from baseline to week 12 differed between the KD and SMD groups. One KD participant had a missing baseline HOMA-IR and fasting glucose measurement (n = 59 for these outcomes). For fasting insulin, the model was fitted on natural logarithm–transformed values because of skewed distribution. Menstrual regularity was analyzed as a binary outcome (1 = regular, 0 = non-regular) using a linear probability model. KD, ketogenic diet; SMD, simulated Mediterranean diet; SE, standard error. *p < 0.05, **p < 0.01, ***p < 0.001, ****p < 0.0001.

## Supplementary Table S3. Post-hoc Power Analysis for the Primary and Key Secondary Outcomes

| Outcome | Effect size | n (KD/SMD) | α (two-sided) | Achieved power |
| --- | --- | --- | --- | --- |
| Menstrual regularity restoration (primary outcome) | Cohen’s h = 0.86 | 30/30 | 0.05 | 91.7% |
| Menstrual recovery among participants with baseline amenorrhea | Cohen’s h = 1.35 | 13/15 | 0.05 | 93.0% |
| Change in HOMA-IR | Cohen’s d = 0.55 | 29/30 | 0.05 | 54.1% |
| Change in body weight | Cohen’s d = 1.81 | 30/30 | 0.05 | > 99.9% |

*Notes:* Post-hoc power was calculated from the observed between-group effect sizes at a two-sided significance level of 0.05. Cohen’s h was used for proportions (96.7% vs. 66.7% for menstrual regularity; 92.3% vs. 33.3% for amenorrhea recovery) and Cohen’s d for continuous outcomes (KD −6.25 ± 4.88 vs. SMD −3.11 ± 6.38 for HOMA-IR change; KD −16.03 kg vs. SMD −6.03 kg for body weight change). The achieved sample size provided adequate power (> 80%) for the co-primary outcome of menstrual regularity and for the large anthropometric effects, whereas power for the between-group difference in HOMA-IR change was limited by the large within-group variability of this marker.

## Supplementary Table S4. Representative Daily Menus and Macronutrient Composition of the Two Dietary Interventions

| Meal | Ketogenic Diet (KD) | Simulated Mediterranean Diet (SMD) |
| --- | --- | --- |
| Breakfast | 2 boiled eggs; ½ avocado; unsweetened soy milk (200 mL); black coffee or tea (no sugar) | Oatmeal (40 g oats) with low-fat milk (200 mL); 1 boiled egg; 1 small apple |
| Lunch | Grilled salmon (120 g); leafy-green salad with olive oil (10 g); steamed broccoli (150 g); a few cherry tomatoes | Cooked brown rice (150 g); steamed sea bass (100 g); stir-fried mixed seasonal vegetables (200 g) with olive oil (5 g); tomato–lentil soup |
| Dinner | Stir-fried lean beef (100 g) with mushrooms and spinach in olive oil (10 g); cucumber–nut salad | Whole-wheat noodles (150 g cooked); chicken breast (80 g); braised tofu with bok choy (150 g); mixed bean salad (50 g) |
| Snack | Walnuts (25 g); cheddar cheese (20 g) | Plain yogurt (150 g); almonds (15 g); blueberries (50 g) |
| Daily total (approx.) | Energy ≈ 1,400 kcal; fat ≈ 112 g (72%); protein ≈ 63 g (18%); carbohydrate ≈ 35 g (10%, <50 g/day) | Energy ≈ 1,360 kcal; fat ≈ 48 g (32%); protein ≈ 61 g (18%); carbohydrate ≈ 170 g (50%) |

Menus are representative examples developed by registered dietitians; actual meal plans were individualized weekly within the same macronutrient targets. KD, ketogenic diet; SMD, simulated Mediterranean diet.
